# Genetic and Environmental Causes of Aggression in the Domestic Cat

**DOI:** 10.3390/vetsci13080754

**Published:** 2026-07-29

**Authors:** Stefano Sartore, Riccardo Moretti, Stefania Chessa, Paola Sacchi

**Affiliations:** Department of Veterinary Science, University of Torino, 10095 Grugliasco, Italy; stefano.sartore@unito.it (S.S.); stefania.chessa@unito.it (S.C.); paola.sacchi@unito.it (P.S.)

**Keywords:** aggression, domestic cat, genetics, environmental causes

## Abstract

Aggression is a behavioral phenomenon observed not only in wild animals but also in companion animals, including the domestic cat. Although feline aggression represents a common welfare and public health concern, its genetic and environmental causes remain not fully understood. The aim of this systematic review was to identify and synthesize the current evidence regarding the genetic and environmental factors associated with aggressive behavior in domestic cats. Original articles published in English between 2005 and 2025 were retrieved from Scopus and PubMed using predefined search strategies. After a two-stage screening and eligibility assessment, 25 studies retrieved though the database searches met the inclusion criteria. One additional eligible study was identified through reference screening, resulting in a total of 26 included studies. No formal risk-of-bias assessment was performed. Findings were synthesized narratively because of the substantial heterogeneity in study design, behavioral assessment methods, and outcome measures. The available evidence indicates that feline aggression is influenced by a complex interaction between genetic predisposition and environmental factors. Associations have been reported with polymorphisms in genes such as OXTR and AR, chromosomal abnormalities, breed, sex, coat color, early-life experiences, owner management, and environmental conditions. Considerable variability in behavioral assessment methods and the absence of a universally accepted classification of feline aggression currently limit direct comparison across studies. Overall, feline aggression should be regarded as a multifactorial trait whose accurate interpretation requires consideration of both inherited and environmental influences.

## Simple Summary

The domestic cat is one of the most widespread companion animals in the world. Aggressive behavior directed toward conspecifics, other animals, or humans is a common welfare concern that may lead to relinquishment, abandonment or, in severe cases, euthanasia. The present systematic review summarizes the current evidence on the genetic basis of feline aggression while also considering the environmental factors that interact with inherited predisposition to shape behavioral expression. The available literature indicates that aggressive behavior should be regarded as a multifactorial phenotype resulting from the interplay between genetic and environmental influences rather than from isolated determinants. However, comparison among studies remains challenging because different forms of aggression are often evaluated using heterogeneous behavioral definitions and assessment methods. A better understanding of these interactions may improve the prevention, clinical management, and welfare of domestic cats.

## 1. Introduction

Aggressiveness in animals represents a fundamental adaptive resource for survival, playing a clearly defined role in territorial defense, access to food resources, and reproductive success. This behavior encompasses a broad spectrum of expression, ranging from ritualized signaling without physical contact [1,2,3] to overt aggression [4], which may result in severe or even fatal consequences [5,6]. Domestication is widely regarded as a mutually beneficial process for both humans and animals [7]. Nevertheless, under specific environmental conditions, even domesticated species such as dogs and cats may exhibit aggressive behaviors reminiscent of their wild ancestors or feral counterparts. This phenomenon is not restricted to partially domesticated species. Rather, across many domesticated taxa, ancestral behavioral traits including aggressiveness appear to have been suppressed rather than eliminated, remaining latent and capable of re-emerging when adaptive demands require it [8,9]. Although the domestication of the dog has been extensively investigated [10,11], comparatively less is known about the domestication of the cat [12]. Current evidence suggests that cat domestication likely began in the Fertile Crescent following the initial interactions between *Felis silvestris lybica* and early agricultural communities [13,14]. From that point forward, the domestic cat progressively diverged from its wild ancestor in terms of docility, behavioral traits, and coat color. Interestingly, unlike many other domesticated species, the domestic cat did not retain pronounced neotenic characteristics [15]. A comparison between canine and feline domestication indicates that the latter progressed more gradually. It was not until the 19th century that cats were widely recognized as companion animals in Europe and the United States [16]. One plausible explanation for this delayed integration relates to the cat’s obligate carnivorous diet, characterized by a limited capacity to digest plant-derived nutrients, a physiological constraint that was fully elucidated only in the 1970s [16]. Only after the development of nutritionally adequate commercial diets did domestic cats become capable of reproducing and thriving without reliance on hunting. This evolutionary and nutritional background may also help explain the persistence of territorial and predatory behaviors in well-fed domestic cats, including the tendency to monitor and defend areas perceived as potential hunting grounds [17]. Because behavioral traits arise from the interaction between inherited predisposition and environmental influences, understanding the genetic basis of feline aggression requires consideration of environmental factors capable of modulating phenotypic expression. A deeper understanding of the genetic and environmental determinants underlying aggressive behavior in domestic cats may contribute to improved welfare outcomes and reduce the risk of abandonment, relinquishment, and euthanasia [18]. Importantly, feline aggression does not represent a single behavioral phenotype. Depending on the context and target, aggressive behavior may be directed toward owners or familiar people, unfamiliar people, conspecifics, other animal species, or occur in specific situations such as handling or veterinary examination. These forms of aggression may arise from different motivational and neurobiological mechanisms and therefore should not automatically be considered equivalent.

The aim of this systematic review was to examine the available evidence on the genetic basis of aggression in the domestic cat, while also considering the environmental factors that may interact with genetic predisposition and contribute to the expression of aggressive behavior.

## 2. Materials and Methods

This systematic review was conducted in accordance with the guidelines outlined in the 2020 state-of-the-art statement of PRISMA (Preferred Reporting Items for Systematic Reviews and Meta-Analyses, checklist in Supplementary Table S1) [19]. The literature search was performed using two electronic databases: Scopus, provided by Elsevier (www.scopus.com; accessed on 26 November 2025), and PubMed, maintained by the National Center for Biotechnology Information (NCBI) (https://pubmed.ncbi.nlm.nih.gov/; accessed on 16 December 2025). The publication period considered ranged from 2005 to 2025, inclusive. Four different combinations of keywords were applied:

1. “cat* behavior” AND “genetic*”;
2. “feline* behavior” AND “genetic*”;
3. “cat*” AND “aggressiv* behavior” AND “genetic*”;
4. “feline*” AND “aggressiv* behavior” AND “genetic*”.

The Boolean operator AND required that all quoted terms appear simultaneously in the search results. The asterisk (*) functioned as a wildcard character, enabling retrieval of all terms sharing the same root. For example, the term “cat*” ensured inclusion of all words beginning with the root “cat” (e.g., cats, category, catabolism, catecholamines, CAT), which explains the initially high number of retrieved records. Similarly, “aggressiv*” captured all lexical variants derived from the root “aggressiv-” (e.g., aggressive, aggressiveness, aggressivity). The search yielded a total of 902 records from Scopus and 1920 records from PubMed. A two-stage screening phase was performed. A first screening phase was conducted to exclude publications unrelated to the objectives of this review. A second screening phase excluded studies published in languages other than English. After these initial screening steps, 18 publications from PubMed and 34 from Scopus were retained. A further eligibility assessment led to the exclusion of review articles, duplicate records, studies focusing on wild cats or other wild felids, and articles addressing exclusively the origin of domestication. Study screening, eligibility assessment, and data extraction were performed by one reviewer and subsequently discussed with the other authors. No major uncertainties requiring formal adjudication arose during the selection process. Following these sequential exclusion steps, 25 topic-relevant scientific articles were selected and included in the present review. Additionally, one study [20] not retrieved through the database search but identified in the reference list of an article used for the introduction [16] was added, resulting in a final total of 26 included studies. The complete list of scientific articles included in this review is presented in Table 1.

Because of the substantial heterogeneity of the included publications in terms of study design, objectives, behavioral phenotypes, and outcome measures, no formal risk-of-bias assessment was performed. This absence was considered when interpreting the overall strength of the evidence.

## 3. Results

### 3.1. Bibliographic Information

For descriptive purposes, the geographical origin of each study was assigned according to the institutional affiliation of the first author, used here as a proxy for the principal research group conducting the study. Based on this classification, 46% of the twenty-six selected articles originated from Europe [21,22,23,24,25,26,27,28,29,30,31,32]; 31% from the United States [20,33,34,35,36,37,38,39]; 11% from Japan [40,41,42]; and 4% from Australia [43], Israel [44], and Turkey [45]. These findings suggest comparatively greater research attention to aggression in the domestic cat within European countries than in other geographic regions. Within Europe, Finland accounted for the highest number of publications (n = 5) [24,25,26,27,28], followed by Spain (n = 2) [21,31]. France [32], Germany [23], Poland [30], and Ukraine [29] contributed one publication each. The predominance of European publications, particularly from Northern European countries, appears consistent with patterns reported in a previous review addressing aggression in domestic dogs published by our group [46]. These findings indicate that the currently available literature is disproportionately represented by European studies, particularly those originating from Finland. The studies included in the present review were published between 2008 and 2025, thus covering nearly the entire time frame considered in the search strategy (2005–2025). The included studies comprised mainly questionnaire-based and observational investigations, together with a smaller number of genetic association studies, experimental behavioral studies, and case reports, highlighting the methodological heterogeneity of the available evidence.

### 3.2. Assessment of Aggression in the Domestic Cat

One of the major methodological challenges emerging from the literature is the absence of a standardized phenotypic classification of feline aggression. Although aggression is frequently discussed as a single behavioral trait, the reviewed studies evaluated different behavioral phenotypes, including aggression toward owners or familiar people, unfamiliar people, conspecifics, dogs, and aggression expressed during handling or veterinary examinations. Furthermore, several studies assessed aggression only as part of broader behavioral or personality dimensions, making direct comparison among studies difficult. In this context, several behavioral assessment tools have been developed. However, many of these instruments remain either unvalidated or only partially validated [47]. More specifically, a survey in 2017 [20] developed and validated the Feline Behavioral Assessment and Research Questionnaire (Fe-BARQ), a structured questionnaire designed to evaluate feline behavior across multiple dimensions, similarly to the already existing C-BARQ (C standing for Canine), first published in 2003 [48]. This instrument integrates various factors, including owner-reported information, direct observations of aggressive behaviors in cats, environmental variables, and expert evaluations of behavioral tendencies across different feline breeds. The aforementioned Fe-BARQ has subsequently been employed in several questionnaire-based studies investigating feline behavior [27,31,36,38]. Other investigations adopted different methodological approaches, including direct behavioral observations, experimental paradigms, clinical assessments, or study-specific behavioral evaluations, depending on their respective study designs. Notably, in two investigations [40,41], a personality questionnaire originally developed for the Akita Inu [49] was adapted for use in cats, highlighting methodological efforts to standardize behavioral assessment across species.

### 3.3. Genetic Causes of Aggression in the Domestic Cat

Aggression, like most behavioral traits, is a complex and multifactorial phenomenon in which genetic factors interact with environmental influences. To date, direct genetic evidence linking feline aggression to specific genomic regions remains limited. The available literature has identified associations involving two candidate genes, together with evidence for the heritability of several behavioral traits and broader genomic signatures related to domestication. A survey in 2014 [34] highlighted the involvement of neural crest-related genes in the evolution of tameness, supporting the hypothesis that genetic mechanisms associated with domestication may also indirectly influence behavioral reactivity, including aggression. A further investigation in 2016 [40] identified a polymorphism in the oxytocin receptor gene (OXTR) associated with the personality trait described as “roughness,” which encompasses aggressive tendencies rather than a specific aggression phenotype. In a subsequent study of the same team [41], an association between a microsatellite region adjacent to the OXTR gene and the same behavioral dimension was reported. Similarly, a recent investigation in 2025 [42] reported an association between polymorphisms in the androgen receptor (AR) gene and stranger-directed aggression in female cats, suggesting a potential role of sex hormone-related genetic pathways in modulating aggression. Furthermore, it was demonstrated by a study in 2019 [26] that several aggression phenotypes are moderately to highly heritable. These findings indicate that aggression should not be considered an isolated trait but rather part of a broader behavioral phenotype influenced by shared genetic architecture. Overall, the available evidence suggests that multiple genetic loci and chromosomal variations contribute to the expression of aggressive behavior in domestic cats, reinforcing the concept of aggression as a polygenic and biologically complex trait.

### 3.4. Breed (i), Gender and Neutering (ii), Coat Color (iii)

(i) Breed-related differences in aggressive and associated behavioral traits have been examined in several studies, suggesting that genetic background may contribute to variability in coping style, sociability, and aggression. A first survey in 2009 [21] investigated behavioral differences between Oriental, Siamese, Abyssinian and Norwegian Forest cat kittens using the Open Field Test. Their findings indicated that Norwegian Forest kittens displayed a more active coping strategy when exposed to challenging or novel situations, suggesting breed-related variation in stress responsiveness. Similarly, a different study in 2016 [24] reported significant breed differences in conspecific-directed aggression. In particular, the Turkish Van and Bengal cat breeds exhibited the highest levels of aggression toward other cats. Moreover, the Turkish Van showed the highest levels of aggression toward unfamiliar humans. Consistent with these findings, a new investigation in 2019 [26] identified the Turkish Van as the breed most strongly associated with aggressive tendencies. In a subsequent study from 2021 [27], it was reported that the Turkish Van demonstrated the highest levels of aggression toward humans and the lowest sociability toward other cats among the breeds examined. Breed differences were also highlighted in a 2024 survey [31], who found that Siamese cats were described as more sociable than Persian cats, yet exhibited greater fear of novelty and more pronounced separation-related behaviors compared with European Shorthair cats. Lastly, in a study assessing adaptability to novel environmental conditions in 2023 [29], the Siamese breed was characterized as more prone to aggressive and retaliatory behaviors, with frequent manifestations of jealousy-related responses. Overall, the available evidence supports the existence of breed-specific behavioral profiles, including differences in aggression, sociability, and coping strategies, which may reflect underlying genetic and selection-related factors.
(ii) Sex and neutering status appear to influence the expression of aggressive and fear-related behaviors in domestic cats, although findings are not entirely consistent across studies considered for inclusion in this review. Specifically, a study in 2008 [44] reported that intact females exhibited higher levels of aggressive behavior toward dogs, whereas spayed females were described as more fearful. Similarly, in a 2024 survey-based study [31], it was found that female cats displayed higher levels of aggression than males toward strangers, owners, and other cats, as well as greater fear responses toward dogs. Furthermore, non-neutered females were less likely to attack familiar cats, while non-neutered males were reported to show reduced object play and lower predatory interest compared with neutered males. In contrast, an investigation in 2025 [22] observed that intact cats were perceived by their owners as more aggressive toward both family members and strangers compared with neutered individuals. However, intact cats were also described as exhibiting a more amicable attitude toward male conspecifics. Lastly, a 2024 study conducted on a colony of free-roaming domestic cats [39] observed that same-sex individuals displayed fewer agonistic interactions when food resources were spatially dispersed, highlighting the interaction between social structure, sex, and environmental conditions in modulating aggressive behavior.
(iii) Coat color has also been examined as a potential correlate of aggression. A 2016 investigation [35] found that tortoiseshell, calico, and torbie (i.e., tortoiseshell–tabby) females scored higher for human-directed aggression than females of other coat patterns. Additionally, black-and-white males were reported to exhibit aggressive behaviors more frequently than males of other coat color. Consistent with these findings, it was noticed in a survey in 2012 [33] that, according to survey respondents, tricolor cats were most frequently described as aloof and intolerant. Moreover, a recent survey [22] reported that tabby-patterned cats were rated as more aggressive than non-tabby individuals. Collectively, these findings suggest that coat color and pattern, whether through genetic linkage, sex-linked inheritance, or perceptual bias, may be associated with differences in reported aggressive behavior in domestic cats. Overall, these findings suggest that sex, reproductive status, coat coloration, and social context may all contribute, individually or interactively, to the variability observed in feline aggressive behavior.

### 3.5. Behavior Problems in Kittens

Early-life experiences appear to play a crucial role in shaping behavioral outcomes, including the development of aggressive tendencies in domestic cats. A first study in 2017 [25] investigated the effects of early weaning (defined as permanent separation from the mother prior to the age at which it would naturally occur) on feline behavior. Their findings indicated that weaning before eight weeks of age significantly increases the risk of aggressive behaviors, although it does not appear to affect fear-related behaviors. In contrast, kittens weaned after 14 weeks of age were less likely to exhibit aggression and stereotypic behaviors, suggesting a protective effect of prolonged maternal contact. Another survey in 2008 [44] reported that a less aggressive behavioral outcome in cats was not associated with whether the cat was adopted before or after a dog within the household. Rather, reduced aggression was linked to experiencing the first interspecific interaction within the first six months of life, highlighting the importance of early socialization timing. Similarly, it was found in 2025 [22] that kittens requiring hand-rearing by owners exhibited higher levels of aggression compared with those raised by their mothers. Overall, these findings emphasize the significance of early maternal care, appropriate weaning age, and timely social experiences in modulating the risk of aggressive behavior in domestic cats.

### 3.6. General Factors Influencing Aggressive Behavior

Several environmental and management-related factors have been shown to influence the expression of aggressive behavior in domestic cats. Firstly, a 2014 survey [43] demonstrated that gentle stroking combined with soft vocalization exerted positive effects on both behavior and health in a group of cats admitted to an animal shelter. These findings suggest that low-stress human interaction may mitigate behavioral distress, potentially reducing aggression in confined environments. In a study conducted on indoor domestic cats in 2024 [38], a positive correlation between aggression displayed during veterinary examinations and the presence of behavioral problems at home was identified, including aggression toward owners and unfamiliar individuals. This association indicates that stress-related reactivity in clinical settings may reflect broader behavioral dysregulation. Another investigation in 2023 [45] studied the effects of the absence of a familiar or socially bonded human figure and reported that such conditions may trigger a cluster of undesirable behaviors collectively referred to as Separation-Related Problems, which can include manifestations of aggression. Additionally, a survey in 2024 [32] highlighted potential behavioral risks associated with selective breeding practices that prioritize aesthetic traits over welfare considerations. Such selection strategies may inadvertently increase the prevalence of maladaptive behaviors, including aggression. Owner-related factors were also implicated. An investigation in 2024 [31] reported that previous owner experience with cats positively influenced feline sociability and was associated with a lower prevalence of problematic behaviors, including aggression toward people and other companion animals. Lastly, a 2022 study [37] found that compounds present in certain plants can induce a euphoric-like state in cats, which may contribute to a reduction in aggression and social conflict. Collectively, these findings underscore the multifactorial nature of feline aggression and emphasize the importance of environmental enrichment, responsible breeding practices, and informed ownership in promoting behavioral stability and welfare.

## 4. Discussion

Although this review considered both genetic and environmental factors associated with feline aggression, its primary objective was to summarize the current evidence regarding the genetic architecture of aggressive behavior while considering environmental factors that interact with inherited predisposition to shape phenotypic expression. This perspective reflects the multifactorial nature of behavioral traits, which arise from the interaction between inherited predisposition and environmental influences rather than from isolated genetic or environmental determinants. Overall, the available evidence indicates that feline aggression should be regarded as a complex behavioral phenotype resulting from the interplay of multiple biological and environmental factors. An important finding emerging from this review is the considerable heterogeneity of the current literature regarding the definition of and assessment of feline aggression. Among the 26 included studies, only a limited number of studies systematically distinguished multiple aggression phenotypes, such as owner-directed, stranger-directed and conspecific-directed aggression, whereas many studies evaluated aggression as a single behavioral trait or incorporated aggressive tendencies with broader personality dimensions. Furthermore, different behavioral assessment tools, study designs, and outcome measures were employed across studies, making direct comparisons difficult. Consequently, caution is required when interpreting the literature or attempting to generalize conclusions regarding “feline aggression” as a single, homogeneous behavioral phenotype. The development of standardized phenotypic definitions and validated behavioral assessment methods would substantially improve the comparability of future studies.

The genetic evidence currently available also requires careful interpretation. Although associations have been reported between aggression-related traits and polymorphisms in genes such as OXTR and AR, together with evidence for moderate heritability of several behavioral traits, these findings should not be interpreted as demonstrating direct genetic determinants of aggression. Rather, they indicate that aggressive behavior is likely influenced by multiple genetic loci acting together with environmental factors throughout behavioral development. Similarly, breed, sex, and coat color should be regarded as phenotypic correlates that may reflect underlying genetic influences, but they are also potentially affected by environmental conditions, owner perception, breeding practices, and population structure. Consequently, these associations should be interpreted with appropriate caution.

The present review also highlights several limitations of the currently available evidence. Most included studies were based on owner-completed questionnaires or observational designs, whereas relatively few experimental or mechanistic investigations were identified. Although owner-reported behavioral assessment represents a valuable approach for large-scale behavioral studies, it is inherently susceptible to reporting and interpretation bias. Moreover, differences in study design, behavioral assessment methods, and aggression phenotypes further limited direct comparisons across studies and precluded quantitative synthesis. An additional limitation of the available literature is the limited evidence regarding age-specific mechanisms of aggression beyond early-life development. No formal assessment of study quality or risk of bias was performed given the substantial methodological heterogeneity, and this should be considered when interpreting the conclusions of the present review. Lastly, this review has some further limitations that should be acknowledged. The literature search was restricted to Scopus and PubMed and included only English-language publications. Although these databases represent the principal sources of indexed biomedical and veterinary literature, potentially relevant studies indexed elsewhere or published in other languages may therefore not be represented in the present review. Furthermore, the search strategy was intentionally designed to identify studies investigating the genetic basis of feline aggression while also capturing evidence regarding environmental factors interacting with inherited predisposition. Consequently, studies focusing exclusively on environmental determinants without reference to behavioral genetics may have been underrepresented.

The domestication of the cat followed a distinct evolutionary trajectory from that of the dog, both in terms of timing and selective pressures. Consequently, behavioral comparisons between the two species should be made with caution. Although domestic cats are widely recognized as domesticated animals, according to several authors they retain a greater proportion of ancestral behavioral characteristics than dogs, particularly regarding territoriality, predatory behavior, and social independence [16]. This evolutionary background may also contribute to unrealistic owner expectations regarding normal feline behavior, increasing the likelihood that species-typical behaviors are perceived as behavioral problems. A better understanding of normal feline behavioral biology therefore represents an essential component of both behavioral prevention and clinical management.

From a practical perspective, the findings summarized in this review reinforce the importance of considering both genetic predisposition and environmental influences when evaluating aggressive behavior in domestic cats. For veterinarians, this implies that behavioral assessment should extend beyond the immediate clinical presentation and include the animal’s early-life history, living environment, and management practices. For owners, increased awareness of normal feline behavior, appropriate socialization, and environmental enrichment may contribute to reducing the risk of behavioral problems and improving animal welfare. Although several management strategies, including environmental enrichment, owner education, and responsible breeding, are biologically plausible and supported by the available literature, the current evidence remains largely observational. Consequently, these recommendations should be interpreted as informed strategies rather than interventions supported by a strong body of experimental evidence.

Future research should prioritize the adoption of standardized definitions and assessment methods for feline aggression, allowing more reliable comparisons among studies and facilitating the identification of biologically meaningful behavioral phenotypes. Greater integration of behavioral, genetic, and environmental data, together with longitudinal and mechanistic studies, will be essential to better characterize gene–environment interactions underlying aggressive behavior. Taken together, these findings reinforce the importance of adopting an integrated genetic and environmental perspective when investigating the biological basis of feline aggression.

## 5. Conclusions

Aggressive behavior in the domestic cat should be regarded as a multifactorial phenotype resulting from the interaction between genetic predisposition and environmental influences rather than from isolated determinants. The available evidence suggests that several genetic factors, including specific polymorphisms and heritable behavioral traits, may contribute to individual variability in aggression. However, their effects are likely modulated by environmental conditions, early-life experiences, management practices, and owner-related factors. The present review also highlights important limitations of the current literature, including the marked heterogeneity in the definition and assessment of aggression, the predominance of questionnaire-based studies, and the limited number of mechanistic investigations. These factors currently prevent direct comparison among studies and underscore the need for standardized behavioral phenotyping and more robust experimental approaches. Despite these limitations, the available evidence provides a valuable framework for understanding the biological and environmental determinants of feline aggression. A more comprehensive characterization of gene–environment interactions and the adoption of standardized behavioral assessment methods will improve our understanding of feline behavioral disorders and may ultimately contribute to the development of more effective prevention, clinical management, responsible breeding strategies, and improvements in animal welfare.

## Figures and Tables

**Table 1:** Complete list of scientific articles included in the present review, showing first author, publication year, journal, DOI, and progressive number in the reference section. The list is ordered by reference number.

First Author	Publication Year	Journal	DOI	Reference Number
Duffy et al.	2017	Behavioural Processes	10.1016/j.beproc.2017.02.010	[20]
Marchei et al.	2009	Physiology & Behavior	10.1016/j.physbeh.2008.11.015	[21]
Pongrácz et al.	2025	Applied Animal Behaviour Science	10.1016/j.applanim.2025.106806	[22]
Hubka et al.	2015	Cell Tissue Research	10.1007/s00441-014-2059-6	[23]
Vapalahti et al.	2016	Frontiers in Veterinary Sciences	10.3389/fvets.2016.00070	[24]
Ahola et al.	2017	Scientific Reports	10.1038/s41598-017-11173-5	[25]
Salonen et al.	2019	Scientific Reports	10.1038/s41598-019-44324-x	[26]
Mikkola et al.	2021	Animals	10.3390/ani11071991	[27]
Mikkola et al.	2023	JAVMA	10.2460/javma.22.10.0441	[28]
Shevchyk et al.	2023	Scientific Horizons	10.48077/scihor.26(2).2023.9-18	[29]
Szczerbal et al.	2023	Animal genetics	10.1111/age.13343	[30]
Menor-Campos et al.	2024	Journal of Veterinary Behavior	10.1016/j.jveb.2023.12.004	[31]
Morel et al.	2024	Animals	10.3390/ani14071003	[32]
Delgado et al.	2012	Anthrozoös	0.2752/175303712X13479798785779	[33]
Montague et al.	2014	Proc Natl Acad Sci U S A	10.1073/pnas.1410083111	[34]
Stelow et al.	2016	Journal of Applied Animal Welfare Science	10.1080/10888705.2015.1081820	[35]
Wilhelmy et al.	2016	Journal of Veterinary Behavior	10.1016/j.jveb.2016.03.009	[36]
Bol et al.	2022	BMC Biology	10.1186/s12915-022-01369-1	[37]
Gerken et al.	2024	Journal of Feline Medicine and Surgery	10.1177/1098612X231214907	[38]
Solomon et al.	2025	Ethology	10.1111/eth.13564	[39]
Arahori et al.	2016	Journal of Veterinary Behavior	10.1016/j.jveb.2015.07.039	[40]
Arahori et al.	2017	Frontiers in Psychology	10.3389/fpsyg.2017.02165	[41]
Okamoto et al.	2025	PLOS One	10.1371/journal.pone.0324055	[42]
Gourkow et al.	2014	Preventive Veterinary Medicine	10.1016/j.prevetmed.2014.06.005	[43]
Feuerstein and Terkel	2008	Applied Animal Behaviour Science	10.1016/j.applanim.2007.10.010	[44]
Kerman et al.	2023	Behavioural Processes	10.1016/j.beproc.2023.104892	[45]

## Data Availability

No new data were created or analyzed in this study.

## Supplementary Materials

The following supporting information can be downloaded at: https://www.mdpi.com/article/10.3390/vetsci13080754/s1, Table S1: PRISMA 2020 checklist.
